# Medical student web-based formative assessment tool for renal pathology

**DOI:** 10.3402/meo.v20.26765

**Published:** 2015-03-31

**Authors:** Vanesa Bijol, Cathryn J. Byrne-Dugan, Melanie P. Hoenig

**Affiliations:** 1 Harvard Medical School, Boston, MA, USA; 2 Department of Pathology, Brigham and Women's Hospital, Boston, MA, USA; 3 Division of Nephrology, Beth Israel Deaconess Medical Center, Boston, MA, USA

**Keywords:** curriculum, quiz, formative assessment, medical education, pathology

## Abstract

**Background:**

Web-based formative assessment tools have become widely recognized in medical education as valuable resources for self-directed learning.

**Objectives:**

To explore the educational value of formative assessment using online quizzes for kidney pathology learning in our renal pathophysiology course.

**Methods:**

Students were given unrestricted and optional access to quizzes. Performance on quizzed and non-quizzed materials of those who used (‘quizzers’) and did not use the tool (‘non-quizzers’) was compared. Frequency of tool usage was analyzed and satisfaction surveys were utilized at the end of the course.

**Results:**

In total, 82.6% of the students used quizzes. The greatest usage was observed on the day before the final exam. Students repeated interactive and more challenging quizzes more often. Average means between final exam scores for quizzed and unrelated materials were almost equal for ‘quizzers’ and ‘non-quizzers’, but ‘quizzers’ performed statistically better than ‘non-quizzers’ on both, quizzed (*p=*0.001) and non-quizzed (*p=*0.024) topics. In total, 89% of surveyed students thought quizzes improved their learning experience in this course.

**Conclusions:**

Our new computer-assisted learning tool is popular, and although its use can predict the final exam outcome, it does not provide strong evidence for direct improvement in academic performance. Students who chose to use quizzes did well on all aspects of the final exam and most commonly used quizzes to practice for final exam. Our efforts to revitalize the course material and promote learning by adding interactive online formative assessments improved students’ learning experience overall.

The concept of the ‘flipped classroom’ has gained increasing popularity in recent years as medical schools across the country reassess the strategies used to teach core curriculum (1, 2). As the emphasis is shifted away from lecture-centered teaching toward student-centered learning, efforts to encourage inquiry and self-study, and actively engage students in their learning process have gained importance. With ever improving educational technology, web-based formative assessment tools have emerged as valuable resources to improve academic performance and enhance motivation and self-directed learning among the new generation of learners. Multiple studies showed positive impact of formative assessments on final exam performance by increasing students’ active participation, preventing procrastination, and providing immediate feedback on their performance. Online administration of formative assessments has great advantages, including easy access and availability, interactive features, effective use of images, immediate and individualized feedback, and automated scoring (3–6). Formative assessment can be very helpful to both students and educators in identifying deficiencies and allowing for timely interventions (6, 7).

In this study, our aim was to explore the value of formative assessment using kidney pathology practice quizzes in our renal pathophysiology course. Students were given unrestricted access to an online kidney pathology quiz system. Our goal was to assess the impact of our intervention on final exam performance, observe the pattern and frequency of quiz use, and to survey the students’ satisfaction with the quizzes at the end of the course. Our particular interest was to assess the difference in final exam performance on quizzed and non-quizzed materials as well as differences in performance between students who did and did not use the quizzes. We hypothesized that the quizzes would have a direct positive impact on students’ academic performance and satisfaction with our course.

## Methods

### The context

Kidney pathology at Harvard Medical School is an integral part of a 3-week-long Renal Pathophysiology course. The kidney pathology curriculum has been taught with didactic lectures and laboratory sessions. The lectures include the review of normal kidney histology and ultrastructure and the mechanisms and pathology of glomerulonephritis and proteinuric renal disease; these topics are further enforced during the laboratory sessions, in addition to material on tubulointerstitial and vascular diseases. Annotated and labeled handouts are provided with all lectures. The participants are second-year medical and dental students (total *n*=161, of which 126 are medical and 35 are dental students). Our study was approved by the Harvard Human Research Protection Program Institutional Review Board (protocol IRB13-3149).

### Design and implementation

Students had the opportunity for self-assessment using online quizzes designed to serve as an adjunct to pre-existing curricular materials. The tool was created using the commercially available software, Articulate^®^ Quizmaker 13. The questions were developed by the first author, and vetted by a group of reviewers consisting of three renal pathologists, three nephrologists, one nephropathology fellow, and five nephrology fellows; the reviewers assessed the questions for content, appropriate level of difficulty, potential errors, and quality of images; and made suggestions for improvement. Fifty questions were available for self-assessment and organized into five distinct quizzes, delivered to students in synchrony with material covered in class. By the end of the second week of the 3-week-long course, all five quizzes were available to students for the remainder of the course. Questions included images in kidney pathology and ranged in difficulty from simple morphological queries to higher level questions that required students to reconcile patterns of injury with clinical syndromes. The quizzes incorporated questions related to morphology and mechanisms of disease (‘quizzed’ materials); they did not include other topics covered in the course such as acid-base and electrolyte disorders, urologic conditions, or urinary tract infections (‘non-quizzed’ materials).

**Fig. 1:**
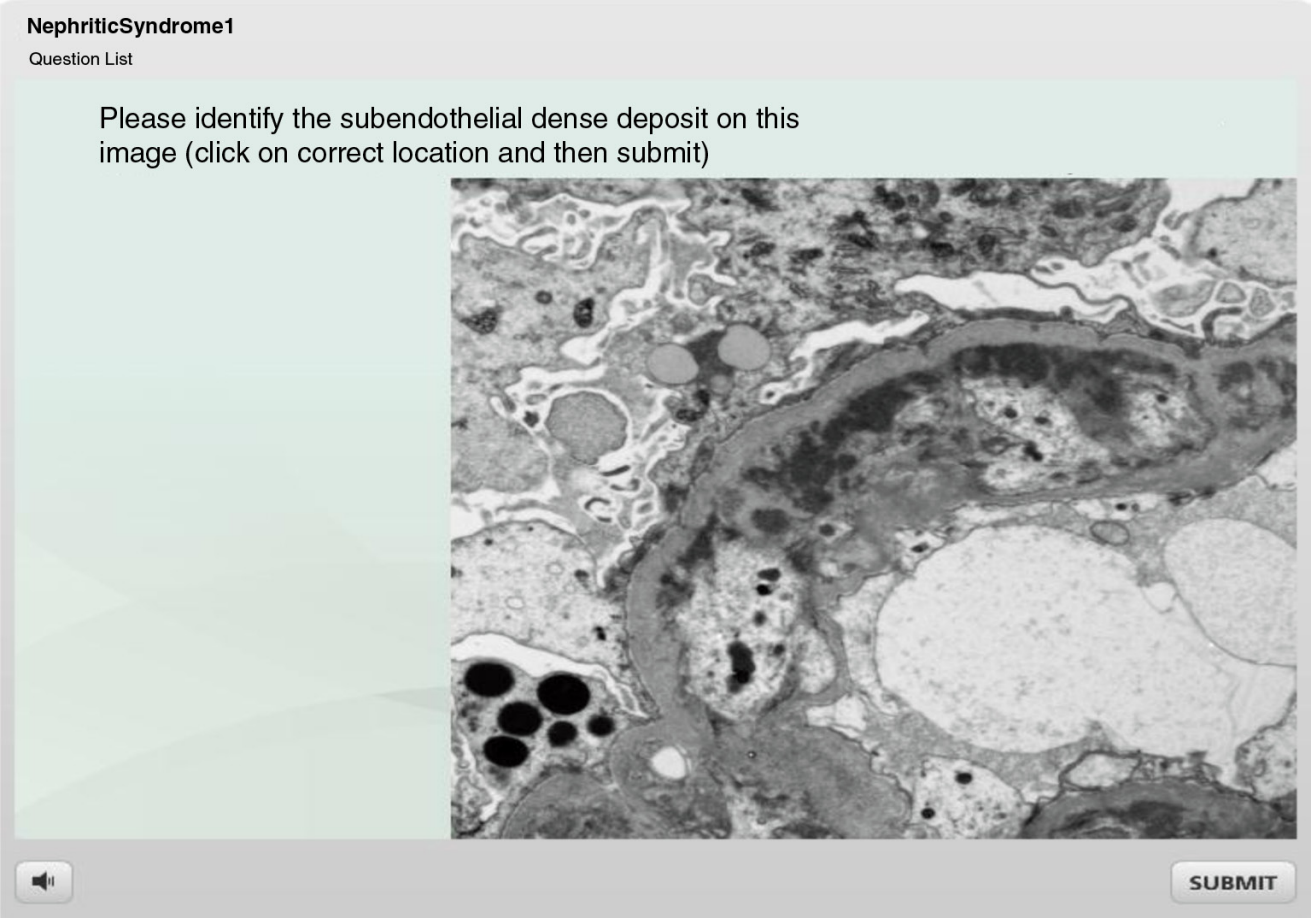
A ‘hot spot’ question format is particularly useful for testing the ability to identify details on microscopic images.

**Fig. 2:**
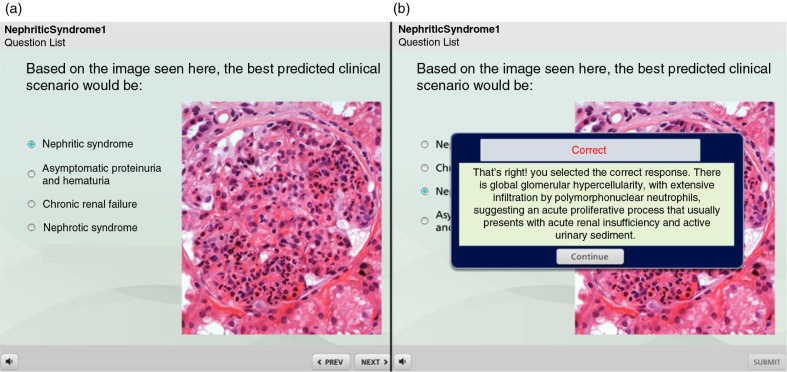
Once the user selects the answer, a feedback screen is displayed to offer the result as correct or incorrect, with brief explanations.

The quizzes were delivered through Articulate^®^ Online, a web-based system equipped with easy publishing features and delivery options, as well as robust usage analysis. Question format varied from multiple-choice to those which asked students to identify a detail (‘hot spot’) on an image, click on a correct image, drop and drag, or mix and match (Fig. 1). Once the user selected an answer, a feedback screen was promptly displayed to offer a brief explanation (Fig. 2). Most questions permitted two attempts to answer, before the system moved on to the next question. After completing the quiz, students could review the questions and explanations and track their own activity and progress.

All students were given individual usernames and passcodes. Access to the quizzes was unrestricted, so students could use quizzes 24 hours a day and 7 days a week, for the duration of the course. There was no limit to the number of times a student could take any of the quizzes. Student participation was optional, and their performance was not individually recorded; however, those students who chose not to use the tool were identified as ‘non-quizzers’, and their performance on the final exam was compared to the students who chose to use the pathology quiz (‘quizzers’). In addition, students’ performance on the pathology content of the exam was compared to their performance on other aspects of the exam, i.e., the ‘non-quizzed’ materials.

### Data collection and analysis

The final examination contained 10 pathology (i.e., morphology and mechanisms of disease) questions and 25 non-pathology (i.e., urology, urinary infections, acid base, and electrolytes) questions. All students took the test at the same time in traditional testing conditions.

Students’ feedback was obtained using an anonymous general course evaluation survey delivered by the secure medical school survey platform and an additional anonymous survey administered through Survey Monkey^TM^. The latter was distributed via email on the last day of the course, followed by a reminder 5 days later. This survey consisted of six questions and a free-text comment section. The survey questions focused on the students’ impression of the course in general, as well as utilization and detailed ratings of different course resources including lecture handouts, reference books, and quizzes and other web-based material. The students were asked to select their lecture attendance preference, which allowed us to compare the resource ratings between students who preferred to attend live lectures versus those who preferred to watch recorded lectures at home. The data were analyzed by Student's *t-*test.

## Results

### Quantitative analysis of the material usage

The five quizzes were viewed a total of 1,487 times by up to 133 unique visitors (82.6% of all students) during 3 weeks. The frequency with which students took the quizzes was the greatest the day before the final summative exam, with the total of 631 attempts (average 126 per quiz) and up to 92 unique visitors (57.1% of all students). This exceeded the usage on any other day by more than a factor of 10.

Students repeated interactive and challenging quizzes more often than those comprised of lower-level multiple-choice questions, even when they obtained similar average scores (Table 1).

**Table 1:** Three different quizzes are compared; one with multiple-choice type questions only, and the other two with a mix of interactive and multiple-choice questions

	Total			Day before exam		
	
Quiz type	No. of views	No. of unique users	Avg. score	No. of views	No. of unique users	Avg. score
Quiz 1 (Multiple-choice)	301	133	78	140	92	83
Quiz 2 (Interactive easy)	319	131	78	157	92	83
Quiz 3 (Interactive and challenging)	365	130	62	188	90	63

Of 161 students, 28 (17.4%) did not use the quiz tool. This group of ‘non-quizzers’ showed similar performance on the final exam for both, quizzed and non-quizzed topics. Interestingly, those students that used pathology quizzes (‘quizzers’) also demonstrated similar performance for quizzed and non-quizzed topics; however, they performed statistically significantly better than ‘non-quizzers’ on the final exam, for both quizzed (*p=*0.001) and non-quizzed (*p=*0.024) topics, respectively (Table 2). We found no statistical difference in usage or final performance between the dental and medical students; 9 of 35 (25%) dental students were non-quizzers, as compared to 19 of 126 (15%) medical students (*p=*0.2), and the overall performance was comparable with average scores 76.72 and 79.04, respectively (*p=*0.20).

**Table 2:** Final examination scores for pathology and non-pathology questions, for students that used (‘quizzers’) and did not use (‘non-quizzers’) the online pathology quiz learning tool

	‘Quizzed’ content mean % (±SD)	‘Non-quizzed’ content mean % (±SD)	*p*
‘Quizzers’ (*n*=133, 82.6%)	79.66 (±8.35)	78.51 (±10.12)	0.317
‘Non-quizzers’ (*n*=28, 17.4%)	73.21 (±12.74)	73.37 (±14.17)	0.967
*p*	0.001	0.024	

### Quantitative analysis of the survey

Of 128 (79.5% of all) students that responded to the general course survey question to rate the pathology portion of the course, 85.9% rated it as excellent or very good, 8.6% as average, and 5.5% below average or poor. The quizzes were evaluated as very useful learning tools by 82.5% of student survey responders. Regarding the question ‘Do you think online quizzes improved the kidney pathology experience in this course?’, 89.0% students responded favorably, whereas 5.0% answered ‘No’, and 6.0% as ‘Not applicable’.

**Fig. 3:**
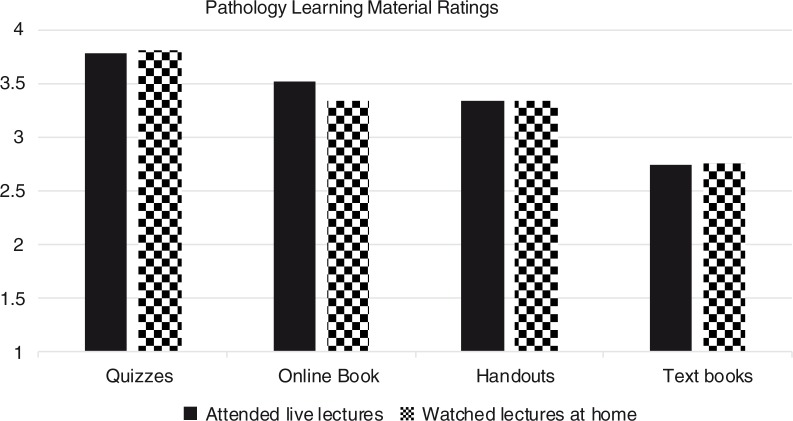
Rating of the offered learning material, from poor (1) to outstanding (4), separated by lecture attendance habits.

In total, 58 students responded to an additional online survey (36.0% overall response rate); of those, 55 reported that they attended lectures (*n*=28) or watched recorded lectures at home (*n*=27). There was no significant difference between these two groups of students with respect to their ratings of course material (Fig. 3). Also, 55 students (96.5%) noted that they would like similar online resources in other courses, whereas 2 students (3.5%) said they had no preference with regard to this matter.

### Qualitative analysis of the survey

The general course survey also included 79 comments regarding the renal pathophysiology course in general; we coded 138 individual entries and grouped them into three large theme groups, including the perceived lecturers’ enthusiasm and positive energy, lecture organization, and the variety and quality of different course resources. About 42% of the comments were directed to the course resources; the students generally liked the availability of different learning formats – from lectures, small groups, and online materials including quizzes. About a third of these comments were specifically directed to quizzes, and they were all very positive. From the comments, it was clear that students were very familiar with the use of quizzes from previous experiences, and they preferred the quiz format with immediate feedback and individualized explanations for all offered choices.

## Discussion

As medical schools across the country consider strategies to invigorate their curricula, many look to the ‘flipped classroom’ to refresh and energize the classroom experience (1, 2, 8). The advent of web-based curricula is a unique opportunity to help achieve this end, aligning with expectations of modern, technologically empowered learners. Active student-centered learning complimented by subsequent group learning has been touted as paramount to develop critical thinking and improve the understanding of fundamental principles. Nevertheless, assessment of these strategies and demonstration of educational gains has lagged behind the reports of the use of creative interactive tools.

The need for new and exciting curricula is particularly important for renal pathophysiology because nearly 80% of surveyed third- and fourth-year students from five medical schools report that renal pathophysiology courses are too complex, lack relevance, or are simply uninteresting (9). This early discouragement may contribute to the dramatic decline in interest for nephrology as a subspecialty that has become evident in recent years (10–12). Renal pathology and specifically the glomerulonephritides are often considered some of the more challenging and confusing concepts for medical students (12, 13). Given the complexity of the content and current students’ dissatisfaction with available offerings, computer-assisted learning is a particularly attractive option to teach renal pathology content. In one study, a computer-assisted learning module on glomerulonephritis was made available to fourth-year medical student volunteers. Following this activity, students were tested on their knowledge of glomerulonephritis. This activity significantly improved student knowledge of glomerular diseases and also decreased the students’ perception of difficulty, in comparison to students who did not use the learning module (matched randomized control group) (13).

Online formative self-assessments in pathology are highly rated in course evaluation surveys and are very effective in promoting student learning (14). It has been observed that formative assessment, if well-designed, can improve student motivation and interest to learn. Timely feedback is essential and unlimited access is a valuable feature that allows for repeated practice, so students can gradually learn the material (3). Student motivation can further be increased by interactive features and by allowing students to keep score or increase the level of difficulty (15). Quizzes with appropriate feedback can help students assess their knowledge and identify areas of weakness, allowing for timely interventions (6, 7).

In our study, we opted to enrich available resources for the entire second-year student class at our medical school to determine if we could improve understanding of renal pathology and limit dissatisfaction and confusion. In addition to traditional textbooks and lecture notes, students were invited to utilize web-based materials, including quizzes. We did not randomize our students to those who used or did not use the quizzes. Instead, the students had a choice to engage in these activities; this increased the likelihood that less motivated and struggling students might fall within the group of ‘non-quizzers’. An interesting and unique aspect in our study was that we were able to compare the final exam performance on quizzed and non-quizzed materials among the two groups of students. As expected, those students who chose to use the online quizzes had better mean scores on the pathology content of the final examination than those who did not utilize the quizzes. Interestingly, these students also achieved higher scores for the final examination on unrelated content, i.e., the material that was not included in quizzes, such as urology, electrolyte and acid base disorders. The average mean between final exam scores for quizzed and non-quizzed materials were almost equal within each student group, but the students that used quizzes performed better on both the quizzed and unrelated topics. Although the use of quizzes did predict the performance on the final examination, our results suggest that students who were sufficiently motivated to use the online quizzes were more likely to do well in all aspects of the final examination. This finding contradicts the concept that retrieval practice, quizzing in particular, directly boosts exam outcomes, and overall academic performance (16). A plausible explanation is instead that students, who take advantage of various learning opportunities, may be more curious and/or motivated, and may have better study habits; these traits may lead to better comprehension and performance outcomes rather than a benefit of the resources themselves (4, 17). The students who are better ‘expert learners’ take a greater responsibility in their achievement outcomes, while actively seeking out opportunities to learn and master self-regulated learning (18). Based on our experience in this study, we also concluded that the prolonged availability of quizzes without structured or restricted access resulted in significantly higher use of quizzes just before the final exam than any period during the course. This implies that the students were not using the formative assessment to facilitate the learning processes, but rather to test their knowledge in preparation for the final exam. This pattern of usage has been described by others (19, 20). Therefore, our goal to facilitate the flipped classroom and invigorate large group learning by offering the quizzes was not realized. An alternative possibility is that the students were overwhelmed with other aspects of the course, as it is still organized as a traditional curriculum that potentially does not allow enough time for self-study, so students cram with quiz exercises just before the final exam. One could also argue that our students were accustomed to more traditional learning strategies and have not developed the culture of self-regulated learning.

Although we were not convinced that our quizzes made a significant difference in knowledge acquisition and the students did not utilize the quizzes to facilitate the flipped classroom as we had hoped, nearly 90% of our survey responders thought the quiz improved their experience in pathology course and more than 95% felt there should be similar resources available in other courses. The students appeared to favor online resources, particularly quizzes, over traditional textbooks, as evidenced by both the high usage and the favorable comments on our surveys. We noted that the students spent more time on quizzes that were interactive and more challenging, than those made of only multiple-choice questions or non-challenging questions. As observed by others, capitalizing on the advantages of computation technology offers a much greater chance of developing an effective and well-received teaching tool (6). Interactive features, video vignettes, links to additional resources of knowledge, ‘pop-up’ windows containing definitions or other important information can all be integrated within the system (21).

In this study, we also showed that there was no difference in resource ratings among students who participated in live lectures versus those who watched recorded lectures. As previously reported, these attendance habits are primarily led by time-saving features of video accelerating technology; in addition, students reported that they were able to better focus and explore additional information when viewing recorded lectures (22). Although in some reports, the correlation between lecture attendance and academic outcomes may reach statistical significance, the link is rather weak and depends on availability of recorded lectures and alternative sources that students may substitute for lectures and still achieve good learning outcomes (23). In this regard, one limitation of our study was that our surveys were anonymous, and we were not able to compare the final exam results between students who attended live lectures with those who watched recordings. However, we were able to show that the attendance habit is not associated with preferences toward the use of traditional textbooks, online books, or quizzes.

Although introduction of novel pedagogy and implementation of web-based technology are popular, it is difficult to prove that it makes a difference (24). Here, too, we showed that our new computer-assisted learning tools are popular, but it is not clear that they foster better knowledge retention, curiosity, or learning. In fact, the results of our study suggest that ‘expert learners’ perform better independent of the strategies used (18). It is likely, therefore, that ‘flipping the classroom’ and placing the responsibility for self-regulated and self-guided learning into student hands will favor the expert learners. Independent of learning outcomes, efforts to improve accessibility of the curriculum, revitalize the material, and promote student's learning are likely to result in an improvement in students’ perception of the material and their experience overall. Complex subjects such as renal pathology are ideally suited to learning with web-based resources. Our experience in renal pathology is a model for other course work and demonstrates the use of modern and interactive technology to compliment traditional teaching and provide an additional platform for student-teacher interactions (13) and learning (20).
